# Piezoresistive Sensing Performance of Smart Layer in Multi-Material 3D-Printed Reinforced Cementitious Beams

**DOI:** 10.3390/s26103204

**Published:** 2026-05-19

**Authors:** Han Liu, Israel Sousa, Shelby E. Doyle, Antonella D’Alessandro, Filippo Ubertini, Simon Laflamme

**Affiliations:** 1 Department of Civil, Construction, and Environmental Engineering, Iowa State University, Ames, IA 50010, USA; 2 Department of Civil and Environmental Engineering, University of Perugia, Via G. Duranti, 93, 06125 Perugia, Italyantonella.dalessandro@unipg.it (A.D.); filippo.ubertini@unipg.it (F.U.); 3 Department of Architecture, Iowa State University, Ames, IA 50010, USA; 4 Department of Electrical and Computer Engineering, Iowa State University, Ames, IA 50010, USA

**Keywords:** 3D printed concrete, self-sensing, structural health monitoring, smart concrete, hybrid printing, piezoresistive properties

## Abstract

3D concrete printing (3DP) enables automated construction with reduced material waste and enhanced geometric flexibility. However, its structural performance remains sensitive to anisotropy, mix design, and printing parameters, thereby complicating quality control. Self-sensing cementitious materials provide a promising approach by enabling intrinsic strain monitoring during fabrication and service. In this study, a hybrid multi-material printing strategy was developed using a conductive cement-based mix incorporating graphite (G), milled carbon microfibers (MCMF), and chopped carbon microfibers (CCMF), alongside a plain cement-based matrix. Based on percolation analysis, an optimal composition of 2 wt.% G, 0.25 wt.% MCMF, and 0.0625 wt.% CCMF was selected. Reinforced beam specimens were fabricated with the conductive material embedded in either the tensile (bottom) or compressive (top) region, combined with two internal architectures: diagonal infill and solid-base configuration. Four configurations were defined: Pattern 1 (bottom/diagonal), Pattern 2 (bottom/solid-base), Pattern 3 (top/diagonal), and Pattern 4 (top/solid-base). Cyclic three-point bending tests with spatially distributed electrical measurements were conducted to evaluate the electromechanical response in the elastic range. Specimens with the conductive layer located in the tensile region (Patterns 1 and 2) consistently exhibited higher gauge factors than those in the compressive region (Patterns 3 and 4). Pattern 2 exhibited the best sensing performance, with an average gauge factor of 556 and SNR of 31. Across all configurations, SNR decreased with increasing electrode spacing, with reductions of up to 31.0%, demonstrating the effect of current path length on sensing performance.

## 1. Introduction

Additive manufacturing, particularly extrusion-based three-dimensional printing (3DP) of cementitious materials, has emerged as a promising construction technology due to its ability to reduce the amount of required formwork, minimize material waste, accelerate construction, enable the fabrication of complex geometries with improved design flexibility [1,2,3]. Despite these advantages, the structural performance of 3D printed components remains highly dependent on the layering quality that is affected by interlayer bonding, printing path, infill topology, and material variability [4,5,6]. These factors can introduce inherent anisotropy and heterogeneity, which significantly affect load transfer mechanisms and structural reliability, thereby posing challenges for large-scale implementation in civil infrastructure [7,8,9].

To address these challenges, one can implement structural health monitoring (SHM) to provide real-time assessment of structural performance during and after fabrication [10,11,12]. Conventional sensing approaches, including strain gauges [13], fiber-optic sensors [14], acoustic emission [15], and piezoelectric transducers [16], have demonstrated effectiveness in evaluating the condition of concrete elements. However, the deployment of conventional SHM technologies in 3DP applications remains limited due to issues related to sensor integration, durability, wiring complexity, and compatibility with the printing process [17]. These limitations motivate the interest in 3DP self-sensing cementitious materials.

Piezoresistive cement-based composites consist of regular cementitious materials mixed with conductive fillers used to increase the piezoresistive effect that creates measurable changes in electrical resistance upon mechanical strain [18,19,20]. Several types of conductive fillers have been studied, including graphite (G) [21,22], carbon microfibers (CMF) [23,24], carbon nanotubes [25,26], and carbon black [27,28] to form percolating conductive networks within the material. Among these, hybrid systems combining particulate fillers and fibrous inclusions have shown particular promise, as they can leverage multiple conduction mechanisms. For example, G primarily contributes to particle-based conduction and tunneling effects, while carbon microfibers enhance network connectivity through bridging across microcracks and microscale gaps within the heterogeneous conductive network [29,30]. As a result, hybrid conductive systems can improve both sensitivity and stability of the electromechanical response.

Existing research on self-sensing cementitious materials can be broadly categorized into two groups. The first focuses on material-level investigations, including material composition, conductive mechanisms, percolation behavior, and electromechanical response in cast or small-scale laboratory specimens [31]. The second includes emerging studies on additive manufacturing of self-sensing systems, which primarily address printability and basic sensing functionality [24,32,33]. Research efforts in this second group remain largely limited to simplified geometries or non-structural components, and do not fully capture the complexity of load-bearing 3D-printed elements. In particular, the combined effects of printing-induced anisotropy, internal architecture, and sensing-layer placement on the electromechanical response remain insufficiently explored.

While the integration of self-sensing functionality into structurally relevant 3D printed components could accelerate the transition of 3DP elements through monitoring and ultimately quality control and assessment, existing studies remain limited in several key aspects, including the integration of sensing functionality within load-bearing printed elements, the influence of printing-induced anisotropy and internal architecture on electromechanical response, and the spatial variability of sensing performance across structural-scale components [34,35,36,37,38]. Our prior study has demonstrated that 3D printed self-sensing cementitious composites can be embedded within structural components to enable integrated strain monitoring [39]. However, in such systems, the sensing response is governed not only by the intrinsic properties of the conductive material, but also by the location of the sensing material relative to the neutral axis, the printing path, infill topology, the presence of reinforcement, and the spatial distribution of strain under loading. These factors introduce additional complexity in interpreting electrical signals, making it challenging to establish a direct and consistently quantifiable relationship between measured resistance changes and structural response.

This study investigates the integration of self-sensing cementitious layers within 3D-printed reinforced beam specimens, termed smart beams, to enable embedded sensing functionality in load-bearing elements. A conductive Sikacrete-based composite incorporating G, MCMF, and CCMF is optimized through percolation analysis and selectively embedded within either the tensile or compressive regions using a multi-material printing approach. Two internal configurations, including a diagonal infill and a solid-base design, are implemented to assess the effect of structural topology on sensing performance. Cyclic three-point bending tests, combined with spatially distributed electrical measurements and an electromechanical model, are used to characterize the piezoresistive response. The novelty of this study lies in three aspects. First, it advances self-sensing cementitious materials from material-scale demonstrations to structurally integrated, load-bearing 3D-printed elements. Second, it quantifies the coupled effects of sensing-layer placement and printing-induced internal architecture on electromechanical response. Third, it introduces a spatially distributed sensing configuration that enables direct evaluation of sensing performance along the beam length, providing new insights into the relationship between structural behavior and electrical response in additively manufactured cementitious components.

The rest of the paper is organized as follows. Section 2 provides the background on 3D printed self-sensing cementitious specimens including the material properties, the fabrication process, and the derivation of the electromechanical model. Section 3 describes the experimental methodology. Section 4 presents and discusses results from the experimental investigation. Section 5 concludes the paper.

## 2. Background

### 2.1. Materials

Self-sensing cement-based composites are fabricated through the incorporation of conductive fillers into the cementitious matrix. The addition of these fillers imparts piezoresistive properties to the composite, enabling it to function as an intrinsic sensing material capable of transducing mechanical stimuli into measurable electrical signals. In this study, a hybrid conductive network was established using G (Thermo Scientific Chemicals, Milwaukee, WI, USA, APS 7–11 μm, 99%) in combination with MCMF (SGL Carbon C M150-4.0/240-UN)) and CCMF (SGL Carbon C M150-4.0/240-G100).

Both types of CMFs exhibited favorable feedability (i.e., the ability of the fresh cementitious mixture to be continuously and reliably transported into the extrusion system without blockage, segregation, or interruption), uniform dispersion within the matrix, and good compatibility with the cementitious matrix [40]. Morphological observations indicate that both fiber types possess relatively smooth surfaces, occasionally featuring longitudinal grooves characteristic of polyacrylonitrile-derived carbon fibers that are produced through the stabilization and carbonization of polyacrylonitrile precursors. A representative scanning electron microscopy (SEM) image of the CMFs is provided in [40], and their detailed material properties are summarized in Table 1. CCMF is received coated with a glycerin-based sizing, which primarily facilitates fiber dispersion and workability with minimal influence on interfacial bonding. The parameter “filament resistivity” denotes the intrinsic electrical resistivity of a single carbon fiber filament as provided by the manufacturer (SGL Carbon GmbH, Arkadelphia, AR, USA). This property is independent of fiber length, as both CCMF and MCMF are derived from the same precursor material and possess identical filament diameters.

### 2.2. Composite Mixtures

Sikacrete 752-3D, a commercially available one-part micro-concrete composed of Portland cement, fine aggregates, and functional admixtures, was adopted as the binder matrix. Its rheology, tailored for extrusion-based 3D printing, exhibits shear-thinning behavior, rapid structural build-up, and adequate open time, enabling stable filament deposition, interlayer bonding, and compatibility with conductive fillers.

The mixture proportions for each set of specimens are summarized in Table 2, and specimens were labeled using the following convention. The numerical prefix denotes the filler content (wt.%) relative to the Sikacrete mass. To investigate the percolation behavior and strain sensitivity of the conductive system, G was first introduced at contents ranging from 0 to 16 wt.%, following a stepwise doubling sequence (1, 2, 4, 8, and 16 wt.%) to capture the evolution of conductive behavior across the percolation regime. The water-to-binder (w/b) ratio was assigned as 0.21, 0.23, 0.26, 0.29, 0.34, and 0.44 for the 0 G, 1 G, 2 G, 4 G, 8 G, and 16 G mixtures, respectively, which were determined through preliminary mixture optimization [39]. Increasing the w/b ratio with increasing G content was necessary to compensate for its high specific surface area, which increases water demand and alters rheological behavior. This adjustment ensured sufficient flowability for extrusion while maintaining adequate structural build-up after deposition [42].

Based on the percolation analysis of the G system, discussed further in Section 4, the 2 wt.% G mixture was identified as being within the percolation zone, providing a balance between electrical sensitivity and printability, and was therefore selected as the reference composition (2G0CMF). MCMF and CCMF were subsequently incorporated over a range of 0.03125 wt.% to 0.25 wt.% following a similar stepwise scaling approach, as presented in Table 2. For mixtures containing CMFs, a constant w/b ratio of 0.26 was adopted, consistent with the 2G reference mixture, in order to eliminate the effect of fiber incorporation. Plain specimens without conductive fillers (0G0CMF) were also prepared for benchmarking results.

A hybrid composition consisting of 2 wt.% G, 0.25 wt.% MCMF, and 0.0625 wt.% CCMF (denoted as 2G250M62CCMF) was selected as the mixture design for 3D printing in this study. This mixture lies near the percolation threshold of each filler, enabling a stable and well-connected conductive network with enhanced sensitivity, while maintaining sufficient workability and buildability for extrusion-based 3D printing. Higher CCMF contents were not considered, as previous studies have shown that excessive fiber loading can lead to nozzle blockage and compromised printability [24,43].

### 2.3. Fabrication Process

**Figure 1:**
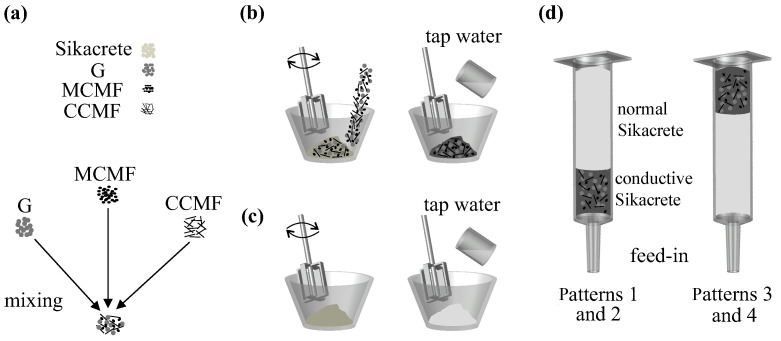
Fabrication process of the cement-based composites: (**a**) conductive fillers; (**b**) preparation of the conductive mix; (**c**) preparation of the normal mix; and (**d**) extrusion cylinder filled with feed-in composite mixtures in two different configurations.

A standardized mixing protocol was adopted to ensure consistent rheological behavior and material dispersion. Two types of mixtures, namely a conductive Sikacrete composite and a normal Sikacrete composite, were prepared as illustrated in Figure 1. For the conductive composite, Sikacrete powder was combined with G, MCMF, and CCMF according to the mix design. All dry constituents were first premixed for 2 min to mitigate particle agglomeration. Subsequently, 50 wt.% of the total mixing water was added, and the mixture was blended using a handheld mixer at 200 rpm for 2 min. The remaining 50 wt.% of water was then introduced to achieve a designed water-to-binder (w/b) ratio, as presented in Table 2, followed by an additional 3 min of mixing at the same speed to ensure adequate homogenization and printability. The normal Sikacrete composite was prepared following the same procedure, excluding the addition of conductive fillers, and using a w/b ratio of 0.21 to account for the absence of high-surface-area additives.

**Figure 2:**
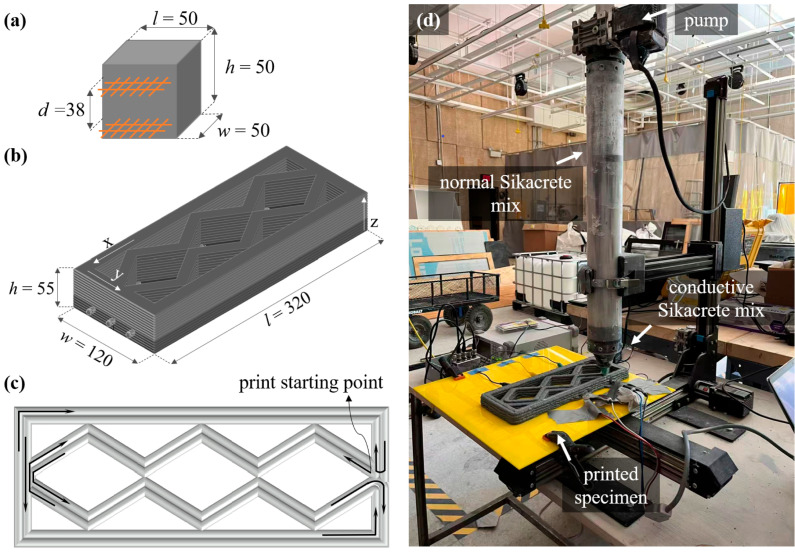
Fabrication of specimens: (**a**) geometry of the cast cubic specimens; (**b**) geometry of the smart beams; (**c**) printing path; and (**d**) overall printing configuration.

A series of cubic specimens (50 × 50 × 50 mm^3^, illustrated in Figure 2a) were fabricated to investigate the percolation behavior and strain sensitivity of the conductive system using the mixture proportions summarized in Table 2. All specimens were produced using a mold-casting approach to allow for the controlled evaluation of the conductive network prior to printing. Each specimen was fabricated using 300 g of normal Sikacrete by placing the mixtures into pre-oiled cubic molds in two stages, with light tamping applied after the first pour to release entrapped air and ensure uniform compaction. Conductive electrodes were embedded during casting, consisting of two parallel copper mesh layers positioned with a fixed spacing interval *D* of approximately 38 mm across the specimen. Sets of three specimens were fabricated under 14 different mix designs for a total of 42 cast specimens.

An extrusion-based commercial 3D clay printer (3D Potter 7) was used for fabricating the smart beams using the selected mix design (2G250M62CCMF). The printer has a resolution in the *X* and *Y* axes of 0.15 mm, and a resolution in the *Z* axis of 0.7 mm. Beam specimens with overall dimensions of 320×120×55 mm^3^ were designed and sliced in Cura into 15 layers with a uniform layer height of 3.66 mm, as shown in Figure 2b. It should be noted that layer height may influence the electromechanical response by affecting interlayer cohesion, internal pore structure, and anisotropy in printed cementitious elements [44,45,46]. The selection of layer height is also governed by factors such as specimen scale, nozzle diameter, and the configuration of the 3D printing system, which affect extrusion stability and geometric fidelity [47,48]. In this study, the adopted layer height was determined based on preliminary trial prints to ensure consistent filament deposition, adequate interlayer bonding, and stable buildability throughout the fabrication process.

The freshly mixed materials were manually loaded into the extrusion cylinder in three consecutive pours, with the loading sequence adjusted to control the placement of the conductive layer. Each pour consisted of approximately 1500 g of fresh material, corresponding to the deposition of five printed layers. Thus, three pours (4500 g in total) were used to complete the 15-layer specimens. For specimens with a bottom conductive layer, the conductive Sikacrete composite was introduced first, followed by two pours of normal Sikacrete. The loading sequence was reversed for specimens designed with the conductive layer at the top, with normal Sikacrete introduced first and the conductive mixture placed in the final pour, as shown in Figure 1d.

A circular nozzle with a diameter of 8 mm was used, and the stand-off distance between the nozzle and the printing platform was maintained at approximately 4 mm. The nozzle movement path is illustrated in Figure 2c. Printing initiates at a predefined corner of the specimen, from which the nozzle first traverses the outer boundary to complete the perimeter of the layer. Subsequently, the nozzle follows a continuous deposition path to construct the internal pattern, advancing along each filament path and translating laterally by approximately one filament width to the adjacent path without interrupting extrusion. This sequence is repeated until all filaments within the layer are completed, with smooth directional transitions at turning points to maintain geometric consistency. Printing was conducted at a constant extrusion rate of 320 mm^3^/s and a platform movement speed of 120 mm/s to ensure stable and uniform material deposition. An image of the printing setup is shown in Figure 2d. The printability of the hybrid conductive mixture was evaluated based on its extrusion stability, filament formation, and buildability during the printing process. Deposited filaments maintained their geometry with minimal deformation, indicating adequate yield stress and structural build-up. The multi-material deposition process was also successfully achieved without notable interfacial instability between the conductive and non-conductive phases. The selected mixture exhibited continuous and uniform extrusion without observable nozzle blockage, filament collapse, or material segregation during the printing process, confirming the proposed mixture design is compatible with extrusion-based multi-material 3D printing and suitable for fabricating structurally relevant components.

Four smart beam configurations were designed by combining two internal pattern types with two conductive layer placements. The internal patterns consisted of a diagonal pattern along the height of the beam, and of a partially solid configuration in which the first six layers were printed with a 100% infill followed by a diagonal pattern for the remaining height (layers 7 to 15). The introduction of a solid base was intended to provide a more uniform and continuous load-transfer region near the bottom of the specimen, thereby reducing geometric-induced variability and enabling assessment of the effect of internal architecture on both structural response and sensing performance.

**Figure 3:**
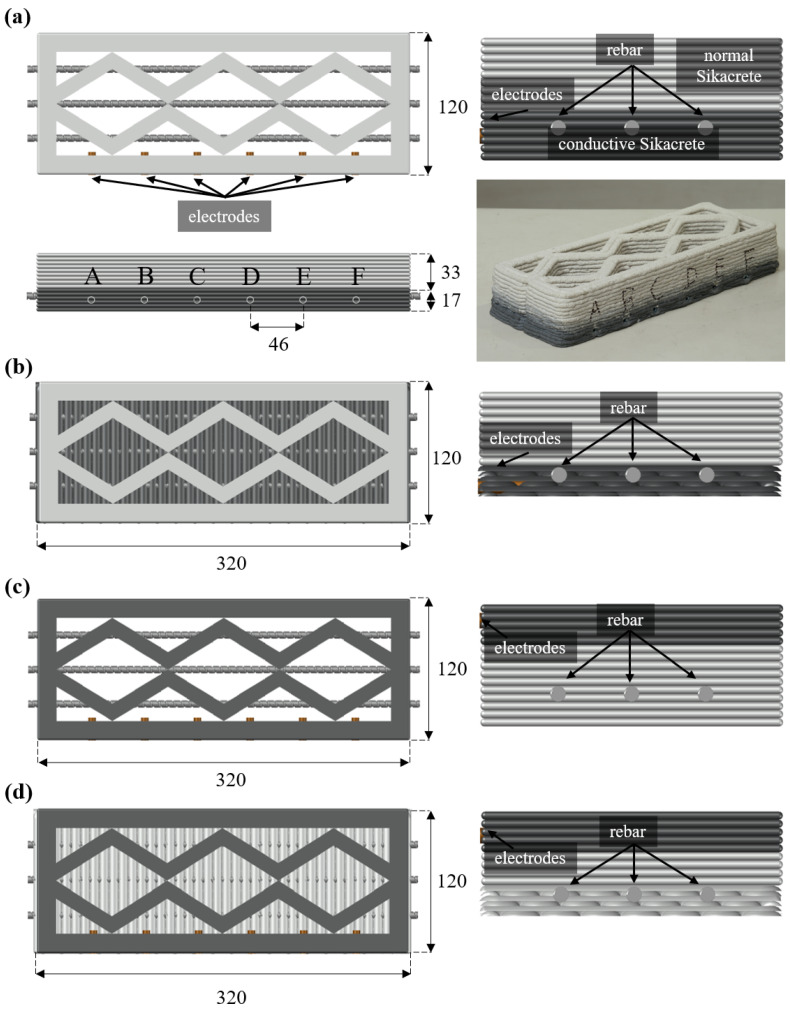
Schematic illustrations of the four sample configurations: (**a**) Pattern 1, including top, side, front, and 3D views; (**b**) Pattern 2, showing top and front views; (**c**) Pattern 3, showing top and front views; and (**d**) Pattern 4, showing top and front views. Units are in mm.

For each internal pattern, two conductive layer arrangements were implemented. In the first arrangement, the conductive material was placed in the bottom six layers (layers 1 to 6), whereas in the second arrangement it was positioned in the top five layers (layers 11 to 15). Accordingly, Pattern 1 corresponds to the diagonal pattern with the conductive layer at the bottom (Figure 3a), Pattern 2 corresponds to the partially solid internal pattern with the conductive layer at the bottom (Figure 3b), Pattern 3 corresponds to the diagonal internal pattern with the conductive layer at the top (Figure 3c). Pattern 4 corresponds to the partially solid internal pattern with the conductive layer at the top (Figure 3d). These four patterns were selected for the independent assessment of the effects of infill topology (diagonal vs. solid base) and sensing-layer location (tension vs. compression) under bending. These designs correspond to a 2×2 factorial design, in which the effects of sensing-layer placement relative to the neutral axis and internal structural topology are independently evaluated.

Six steel drop-in anchors were embedded as electrodes at equal spacing along the beam length and labeled sequentially as A–F (as shown in Figure 3a). For specimens with bottom conductive layers (Patterns 1 and 2), the electrodes were positioned in the third layer, whereas for specimens with top conductive layers (Patterns 3 and 4), the electrodes were embedded in the twelfth layer to align with the conductive region. In addition, three #2 steel reinforcement rebars (diameter 6.35 mm) were placed longitudinally after deposition of the fifth layer to provide a more realistic structural configuration and to ensure elastic behavior under bending during testing. A center-to-center spacing of 30 mm between the longitudinal reinforcement bars was adopted, measured from the center of each bar, to prevent direct contact between the reinforcement and the embedded electrodes. The beam specimens were designed to represent structurally relevant elements, incorporating longitudinal reinforcement and beam-scale geometry to approximate realistic load-bearing conditions. The inclusion of reinforcement and controlled geometry ensures that the measured electromechanical response reflects behavior under representative flexural loading rather than material-scale testing. Following fabrication, all specimens were cured under laboratory conditions for 28 days to promote hydration and ensure stable mechanical and electrical performance.

### 2.4. Electromechanical Model

The electromechanical response of self-sensing cement-based materials is commonly described through their piezoresistive behavior, whereby mechanical deformation induces a measurable change in electrical resistance. For the conductive region between the embedded electrodes, the nominal electrical resistance can be expressed as

(1) $$R_{0}=\rho \frac{D}{A}$$

where ρ is the bulk electrical resistivity of the conductive cementitious composite, *D* is the electrode spacing, and *A* is the effective cross-sectional area normal to the current path. For the present 3D-printed beam specimen, the conductive paths are non-uniform due to the heterogeneous, percolated conductive network and the effect of internal architecture. As such, *A* does not correspond to a strictly geometric cross-section, but is treated as a parameter that is constant for a given pattern configuration in the undeformed state. Under mechanical loading, its incremental variation due to deformation is captured through the differential term dA/A. While this assumption limits direct physical interpretation, it enables a tractable formulation for comparative analysis. Taking the natural logarithm of Equation (1) gives

(2) lnR=lnρ+lnD−lnA

Differentiating Equation (2) yields

(3) $$\frac{dR}{R}=\frac{d\rho }{\rho }+\frac{dD}{D}-\frac{dA}{A}$$

Assuming that the conductive region undergoes uniform longitudinal deformation, isotropic lateral contraction is considered for the transverse dimensions due to Poisson’s effect. Let *b* and *t* denote the width and thickness of the conductive region between the electrodes, respectively. The corresponding transverse strain components are expressed as

(4) $$\frac{db}{b}=\frac{dt}{t}=-\nu \frac{dD}{D}$$

where ν is the Poisson’s ratio of the conductive cementitious material. The cross-sectional area therefore varies with deformation, and its relative change can be written as

(5) $$\frac{dA}{A}=\frac{db}{b}+\frac{dt}{t}=-2\nu \frac{dD}{D}$$

Substituting Equation (5) into Equation (3) gives

(6) $$\frac{dR}{R}=\frac{d\rho }{\rho }+\left(1+2\nu \right)\frac{dD}{D}$$

which shows that the change in resistance arises from both intrinsic resistivity variation and geometrical effects associated with deformation. For small deformations, the infinitesimal form may be approximated by finite increments

(7) $$\frac{\Delta R}{R_{0}}=\frac{\Delta \rho }{\rho }+\left(1+2\nu \right)\frac{\Delta D}{D}=\frac{\Delta \rho }{\rho }+\left(1+2\nu \right)\varepsilon$$

and the fractional change in resistance (FCR) becomes

(8) $$\mathrm{FCR}=\frac{\Delta R}{R_{0}}=\frac{\Delta \rho }{\rho }+\left(1+2\nu \right)\varepsilon$$

Equation (8) represents the local electromechanical constitutive relation of the sensing material. However, for the beam specimens printed in this study, the strain in the sensing region is pattern-dependent because the four configurations differ in the infill topologies, conductive-layer location, and reinforcement layout.

At a first-order approximation, the printed elements are modeled as Euler–Bernoulli elastic beams for interpreting the results. This is a simplification, given the geometry of the specimens—characterized by varying cross-sections along their length and widths comparable to their lengths—as well as the anisotropic behavior of printed cement-based materials. This assumption neglects the effects of material heterogeneity, anisotropy, and printing paths inherent to 3D-printed cementitious specimens. Nevertheless, it provides a consistent framework for capturing the global bending behavior and estimating the average strain in the sensing layer across different configurations, and is therefore sufficient to describe the main physical phenomena. Under three-point bending, the mid-span deflection δ of a simply supported beam subjected to a central point load *P* is given by

(9) $$\delta =\frac{PL^{3}}{48EI}$$

where *L* is the span length, *E* is the effective elastic modulus, assumed to be constant along the beam length as a first-order approximation, and *I* is the effective moment of inertia of the transformed section. The maximum bending moment at mid-span is $M_{\max}=PL/4$, and the curvature (κ) at mid-span is defined as

(10) $$\kappa =\frac{M_{\max}}{EI}=\frac{1}{EI}\cdot \frac{PL}{4}=\frac{1}{EI}\cdot \frac{48EI\delta }{L^{3}}\cdot \frac{L}{4}=\frac{12\delta }{L^{2}}$$

Let *z* denote the vertical coordinate measured from the bottom surface of the beam, $z_{\mathrm{NA}}^{\left(p\right)}$ the neutral-axis location for pattern *p*, and $z_{s}^{\left(p\right)}$ the centroid of the conductive sensing region. The bending strain in the sensing layer is then written

(11) $$\varepsilon_{s}^{\left(p\right)}=\kappa \left(z_{\mathrm{NA}}^{\left(p\right)}-z_{s}^{\left(p\right)}\right)=\frac{12\delta }{L^{2}}\left(z_{\mathrm{NA}}^{\left(p\right)}-z_{s}^{\left(p\right)}\right)$$

where p=1,2,3,4 denotes the beam pattern. For the smart beams Patterns 1 and 2, the centroid of the bottom conductive layer is located at $z_{b}=10.98$ mm, and for Patterns 3 and 4, the centroid of the top conductive layer is located at $z_{t}=45.75$ mm, giving

(12) $$\varepsilon_{s}^{\left(1\right)}=\frac{12\delta }{L^{2}}\left(z_{\mathrm{NA}}^{\left(1\right)}-z_{b}\right)$$

(13) $$\varepsilon_{s}^{\left(2\right)}=\frac{12\delta }{L^{2}}\left(z_{\mathrm{NA}}^{\left(2\right)}-z_{b}\right)$$

(14) $$\varepsilon_{s}^{\left(3\right)}=\frac{12\delta }{L^{2}}\left(z_{\mathrm{NA}}^{\left(3\right)}-z_{t}\right)$$

(15) $$\varepsilon_{s}^{\left(4\right)}=\frac{12\delta }{L^{2}}\left(z_{\mathrm{NA}}^{\left(4\right)}-z_{t}\right)$$

Under three-point bending, the bottom region of the beam is subjected to tension and the top region to compression. Accordingly, the sensing layer experiences tensile strain in Patterns 1 and 2 ($\varepsilon_{s}^{\left(1\right)},\varepsilon_{s}^{\left(2\right)}>0$), and compressive strain in Patterns 3 and 4 ($\varepsilon_{s}^{\left(3\right)},\varepsilon_{s}^{\left(4\right)}<0$), following the sign convention adopted herein.

The neutral-axis location $z_{\mathrm{NA}}^{\left(p\right)}$ varies among the four patterns due to differences in internal architecture and the presence of longitudinal reinforcement. It is determined using a transformed-section approach that accounts for both the cementitious matrix and the steel reinforcement. In general form,

(16) $$z_{\mathrm{NA}}^{\left(p\right)}=\frac{\sum_{i}E_{i}A_{i}z_{i}+\sum_{j}E_{s}A_{s,j}z_{s,j}}{\sum_{i}E_{i}A_{i}+\sum_{j}E_{s}A_{s,j}}$$

where $A_{i}$ and $z_{i}$ are the area and centroid of the *i*th printed subregion, $A_{s,j}$ and $z_{s,j}$ are the area and centroid of the *j*th reinforcing bar, and $E_{i}$ and $E_{s}$ denote the elastic moduli of the cementitious material and steel, respectively.

Substituting the pattern-dependent sensing strain into Equation (8) gives

(17) $${\mathrm{FCR}}^{\left(p\right)}=\frac{\Delta R}{R_{0}}=\frac{\Delta \rho }{\rho }+\left(1+2\nu \right)\varepsilon_{s}^{\left(p\right)}$$

The bending gauge factor for each pattern is then defined as

(18) $$\lambda^{\left(p\right)}=\frac{d({\mathrm{FCR}}^{\left(p\right)})}{d(\varepsilon_{s}^{\left(p\right)})}$$

### 2.5. Pattern- and Electrode-Pair-Dependent Bending Strain

When the electrical response is measured between different electrode pairs, the strain input to the electromechanical model is not unique, because it depends on both the beam pattern and the spanwise location of the selected electrode pair. The strain used for electromechanical analysis is therefore defined as the average bending strain over the conductive region between any electrodes I, and J under pattern *p*.

Let $x_{A}<x_{B}<x_{C}<x_{D}<x_{E}<x_{F}$ denote the longitudinal coordinates of electrodes A-F, measured from the left support, and let $x_{IJ}=x_{J}-x_{I}$ denote the gauge length between electrodes *I* and *J*. Here, the six electrodes are equally spaced at 46 mm intervals and are assumed to be symmetrically arranged about the beam mid-span, independent of the pattern configuration. With s=46 mm, their coordinates are written as

(19) $$x_{A}=\frac{L-5s}{2},\;x_{B}=\frac{L-3s}{2},\;x_{C}=\frac{L-s}{2},\;x_{D}=\frac{L+s}{2},\;x_{E}=\frac{L+3s}{2},\;x_{F}=\frac{L+5s}{2}$$

Under three-point bending, the curvature distribution along the beam is

(20) $$\kappa \left(x\right)=\left\{\begin{array}{cc} \frac{24\delta }{L^{3}}x, & 0\leq x\leq \frac{L}{2}\\ \frac{24\delta }{L^{3}}\left(L-x\right), & \frac{L}{2}\leq x\leq L \end{array}\right.$$

where δ is the mid-span deflection and *L* is the support span. This formulation assumes a perfect bond between the reinforcement and the surrounding cementitious matrix. Possible bond-slip behaviors, especially near the beam ends, are neglected. The average curvature over the sensing length between electrodes *I* and *J* is then

(21) $${\bar{\kappa }}_{IJ}=\frac{1}{x_{IJ}}\int_{x_{I}}^{x_{J}}\kappa \left(x\right)\;dx$$

where the bar in $\left(\bar{\kappa }\right)$ denotes the spatial average over the electrode gauge length between $x_{I}$ and $x_{J}$. The corresponding average bending strain between electrodes *I* and *J* ($\bar{\varepsilon }$) in the conductive sensing layer for pattern *p* is written as

(22) $${\bar{\varepsilon }}_{IJ}^{\left(p\right)}=c_{p}{\bar{\kappa }}_{IJ}$$

where $c_{p}$ is the signed distance between the neutral axis and the centroid of the conductive sensing layer for pattern *p*:

(23) $$c_{p}=z_{\mathrm{NA}}^{\left(p\right)}-z_{s}^{\left(p\right)}$$

Thus,

(24) $$c_{1}=z_{\mathrm{NA}}^{\left(1\right)}-z_{b},\;c_{2}=z_{\mathrm{NA}}^{\left(2\right)}-z_{b},\;c_{3}=z_{\mathrm{NA}}^{\left(3\right)}-z_{t},\;c_{4}=z_{\mathrm{NA}}^{\left(4\right)}-z_{t}$$

Substituting Equation (19) into Equations (20)–(22), the average bending strains for the electrode pairs are obtained as follows.

For adjacent electrode pairs,

(25) $${\bar{\varepsilon }}_{AB}^{\left(p\right)}={\bar{\varepsilon }}_{EF}^{\left(p\right)}=c_{p}\;\frac{\delta }{L^{3}}\left(12L-48s\right)=c_{p}\;\frac{\delta }{L^{3}}\left(12L-2208\right)$$

(26) $${\bar{\varepsilon }}_{BC}^{\left(p\right)}={\bar{\varepsilon }}_{DE}^{\left(p\right)}=c_{p}\;\frac{\delta }{L^{3}}\left(12L-24s\right)=c_{p}\;\frac{\delta }{L^{3}}\left(12L-1104\right)$$

(27) $${\bar{\varepsilon }}_{CD}^{\left(p\right)}=c_{p}\;\frac{\delta }{L^{3}}\left(12L-6s\right)=c_{p}\;\frac{\delta }{L^{3}}\left(12L-276\right)$$

For non-adjacent electrode pairs,

(28) $${\bar{\varepsilon }}_{AC}^{\left(p\right)}={\bar{\varepsilon }}_{DF}^{\left(p\right)}=c_{p}\;\frac{\delta }{L^{3}}\left(12L-36s\right)=c_{p}\;\frac{\delta }{L^{3}}\left(12L-1656\right)$$

(29) $${\bar{\varepsilon }}_{AD}^{\left(p\right)}={\bar{\varepsilon }}_{CF}^{\left(p\right)}=c_{p}\;\frac{\delta }{L^{3}}\left(12L-26s\right)=c_{p}\;\frac{\delta }{L^{3}}\left(12L-1196\right)$$

(30) $${\bar{\varepsilon }}_{AE}^{\left(p\right)}={\bar{\varepsilon }}_{BF}^{\left(p\right)}=c_{p}\;\frac{\delta }{L^{3}}\left(12L-\frac{51}{2}s\right)=c_{p}\;\frac{\delta }{L^{3}}\left(12L-1173\right)$$

(31) $${\bar{\varepsilon }}_{AF}^{\left(p\right)}=c_{p}\;\frac{\delta }{L^{3}}\left(12L-30s\right)=c_{p}\;\frac{\delta }{L^{3}}\left(12L-1380\right)$$

(32) $${\bar{\varepsilon }}_{BD}^{\left(p\right)}={\bar{\varepsilon }}_{CE}^{\left(p\right)}=c_{p}\;\frac{\delta }{L^{3}}\left(12L-15s\right)=c_{p}\;\frac{\delta }{L^{3}}\left(12L-690\right)$$

(33) $${\bar{\varepsilon }}_{BE}^{\left(p\right)}=c_{p}\;\frac{\delta }{L^{3}}\left(12L-18s\right)=c_{p}\;\frac{\delta }{L^{3}}\left(12L-828\right)$$

These expressions show that the strain input to the electromechanical model depends on both the beam pattern, through $c_{p}$, and the selected electrode pair, through its spanwise position relative to the curvature distribution. Accordingly, the pair-dependent fractional change in resistance is written as

(34) $${\mathrm{FCR}}_{IJ}^{\left(p\right)}=\frac{\Delta R_{IJ}^{\left(p\right)}}{R_{0,IJ}^{\left(p\right)}}=\frac{\Delta \rho_{IJ}^{\left(p\right)}}{\rho_{IJ}^{\left(p\right)}}+\left(1+2\nu \right){\bar{\varepsilon }}_{IJ}^{\left(p\right)}$$

and the corresponding pair-dependent gauge factor is defined as

(35) $$\lambda_{IJ}^{\left(p\right)}=\frac{{\mathrm{FCR}}_{IJ}^{\left(p\right)}}{{\bar{\varepsilon }}_{IJ}^{\left(p\right)}}$$

The magnitude of the average bending strain for different electrode pairs is determined solely by their spanwise position relative to the curvature distribution. Therefore, the theoretical ranking of the bending strain magnitude is given by

(36) $$\begin{array}{cc} |{\bar{\varepsilon }}_{CD}| & >|{\bar{\varepsilon }}_{BD}\left|=\right|{\bar{\varepsilon }}_{CE}\left|>\right|{\bar{\varepsilon }}_{BE}\left|>\right|{\bar{\varepsilon }}_{BC}\left|=\right|{\bar{\varepsilon }}_{DE}|\\ \; & >|{\bar{\varepsilon }}_{AE}\left|=\right|{\bar{\varepsilon }}_{BF}\left|>\right|{\bar{\varepsilon }}_{AD}\left|=\right|{\bar{\varepsilon }}_{CF}|\\ \; & >|{\bar{\varepsilon }}_{AF}\left|>\right|{\bar{\varepsilon }}_{AC}\left|=\right|{\bar{\varepsilon }}_{DF}\left|>\right|{\bar{\varepsilon }}_{AB}\left|=\right|{\bar{\varepsilon }}_{EF}| \end{array}$$

To further quantify this relationship, the bending strain is normalized with respect to the CD segment, i.e., $|{\bar{\varepsilon }}_{CD}|=1$. The corresponding normalized strain magnitudes for all electrode pairs are then expressed as

(37) $$\begin{array}{cc} |{\bar{\varepsilon }}_{CD}| & =1.000\\ |{\bar{\varepsilon }}_{BD}\left|=\right|{\bar{\varepsilon }}_{CE}| & =0.884\\ |{\bar{\varepsilon }}_{BE}| & =0.845\\ |{\bar{\varepsilon }}_{BC}\left|=\right|{\bar{\varepsilon }}_{DE}| & =0.768\\ |{\bar{\varepsilon }}_{AE}\left|=\right|{\bar{\varepsilon }}_{BF}| & =0.748\\ |{\bar{\varepsilon }}_{AD}\left|=\right|{\bar{\varepsilon }}_{CF}| & =0.742\\ |{\bar{\varepsilon }}_{AF}| & =0.690\\ |{\bar{\varepsilon }}_{AC}\left|=\right|{\bar{\varepsilon }}_{DF}| & =0.613\\ |{\bar{\varepsilon }}_{AB}\left|=\right|{\bar{\varepsilon }}_{EF}| & =0.458 \end{array}$$

The proposed formulation is intended to establish the relationship between the change in resistance and bending strain and to interpret the spatial variations of the electromechanical response. The consistency between the theoretical strain distribution and the experimentally observed FCR trends across electrode pairs indicates that the model captures the dominant behavior, as discussed in detail in Section 4.

The signal-to-noise ratio (SNR) was employed to quantitatively evaluate signal quality. The SNR measures the relative energy of the signal with respect to the noise and computed as:

(38) $${\mathrm{SNR}}_{\mathrm{dB}}=10\log_{10}\left(\frac{P_{\mathrm{signal}}}{P_{\mathrm{noise}}}\right)$$

where $P_{\mathrm{signal}}$ and $P_{\mathrm{noise}}$ denote the powers of the signal and noise, respectively.

## 3. Experiments

### 3.1. Measurements

Impedance spectra were measured using an LCR meter (Agilent 4263B, 100 Hz–100 kHz sweep, resistance–reactance mode) under two AC excitation amplitudes (0.1 V and 1.0 V) to evaluate the quality of conductive filler dispersion. The frequency range is selected for the response to be dominated by bulk conductive behavior and less by capacitive and interfacial effects [49,50]. Measurements were taken at 28 days of curing to ensure a stabilized hydration state. An AC two-probe configuration was adopted, which is suitable for highly resistive cementitious systems where direct current measurements may be affected by polarization effects [50,51]. Based on the selected measurement configuration and previous research works, embedded copper mesh electrodes were used to improve electrical contact at the electrode–matrix interface and to reduce contact resistance [52].

The obtained impedance spectra were analyzed in two ways. First, the full frequency-dependent response (100 Hz–100 kHz) was examined to assess dispersion quality, frequency stability, and conductive network formation. Second, a characteristic frequency of 1 kHz was selected for quantitative comparison across mixtures, as it provides a balance between minimizing electrode polarization effects and capturing the bulk conductive response associated with the percolated filler network [49,50]. Data acquisition was performed using LabVIEW to monitor potential polarization drift during testing, which may arise from dielectric responses of the cement-based matrix [53,54] or possible piezoelectric effects [55,56]. All measurements were conducted under controlled laboratory conditions with consistent temperature and humidity.

### 3.2. Flexural Tests

**Figure 4:**
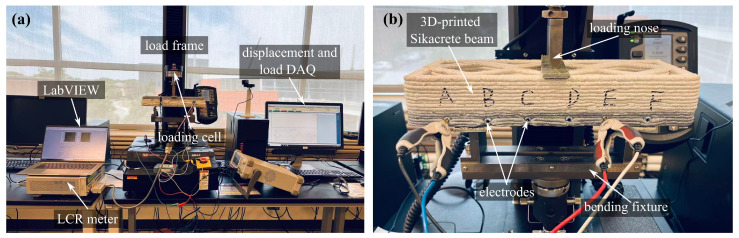
Experimental setup showing: (**a**) overall experimental configuration; and (**b**) close-up view of the flexural bending setup.

The electromechanical response of the self-sensing Sikacrete beam was evaluated under controlled flexural loading using a three-point bending configuration. Figure 4a shows the overall experimental setup. Testing was performed using an Instron 5944 universal testing frame equipped with a 1 kN (Series 2580) load cell. The testing machine utilized here has been extensively calibrated and validated for strain measurements, and given the small size of specimens and the axial loading configuration, it was assumed that the strain or force applied by the testing machine was that experienced by the materials. Beam specimens were supported over a 200 mm span, and a concentrated load was applied at midspan through the loading nose. To prevent electrical shorting during simultaneous mechanical and resistance measurements, fiberglass insulating plates were inserted between the specimen and both the loading nose and the support rollers, as shown in Figure 4b.

Loading was applied in a cyclic triangular waveform with progressively increasing amplitude. Two initial cycles were conducted up to 150 N at a loading rate of 30 N/s, followed by two cycles reaching 300 N at 60 N/s. The final two cycles were conducted at a peak load of 600 N at 120 N/s. This loading protocol was designed to remain within the elastic regime to ensure repeatability and to isolate the intrinsic piezoresistive response of the conductive network. While microcracking can significantly influence conductive pathways and enhance sensitivity, it also introduces irreversible and path-dependent effects that complicate interpretation. The present results therefore represent baseline sensing behavior independent of damage-related effects.

Prior to the cyclic protocol, a preload of 20 N was applied to ensure stable contact conditions and eliminate seating-related compliance effects in the fixture assembly. The mechanical response was acquired continuously at 100 samples/second (S/s), including both applied load and mid-span displacement. In parallel, spatially distributed electrical measurements were obtained at 10 S/s across adjacent electrode pairs (AB, BC, CD, DE, and EF). Electrical resistance was measured independently for each segment using an LCR meter operating in resistance–reactance (R–X) mode, with data acquisition automated through a LabVIEW interface. This synchronized measurement scheme enabled direct correlation between flexural deformation and localized resistance variation along the beam length.

## 4. Results and Discussion

### 4.1. Particle Distribution

**Figure 5:**
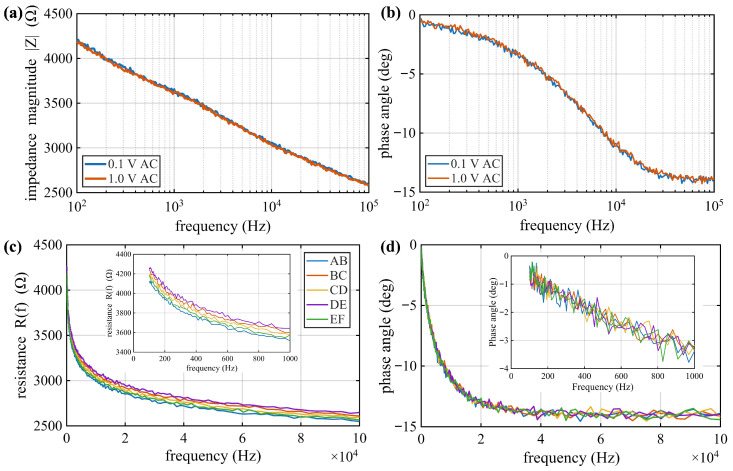
Impedance-based evaluation of conductive filler dispersion: (**a**) impedance magnitude under 0.1 V and 1.0 V excitation; (**b**) phase angle spectra showing negligible amplitude dependence; (**c**) resistance R(f); and (**d**) phase spectra cross adjacent segments (AB–EF).

The impedance magnitude and phase angle spectra obtained from a representative electrode pair (e.g., C–D) in the cast cubic specimens under two excitation amplitudes are presented in Figure 5a,b. The responses exhibit near-complete overlap across the entire frequency range from 100 Hz to 10 kHz, indicating negligible amplitude-dependent variation. The impedance magnitude remains in the low-kΩ range (approximately 4 to 4.5 kΩ at low frequency) and decreases gradually with increasing frequency, while the phase angle decreases from near-resistive behavior to a modest capacitive response (approximately 0 to −15°), associated with interfacial polarization and dielectric effects within the cementitious matrix [50,57]. No measurable amplitude-dependent distortion or nonlinear behavior is observed within experimental uncertainty, confirming that the conductive network operates within a stable linear regime.

More importantly, impedance responses measured across adjacent electrode segments (AB, BC, CD, DE, and EF) exhibit low spatial variability in both resistance magnitude and frequency-dependent characteristics, as shown in Figure 5c,d. The resistance spectra R(f) across different segments remain closely clustered, with only minor variations in magnitude (within approximately 5 to 10%), while the spectral slope and phase evolution show consistent trends. The characteristic transition behavior in the phase response also exhibits minimal deviation among segments. Such spatial consistency indicates that conductive fillers are well dispersed along the beam length, forming a continuous and homogeneous percolated network. The high repeatability observed across repeated frequency sweeps further supports reliable electrode embedment and stable electrical contact throughout the specimen.

### 4.2. Percolation

**Figure 6:**
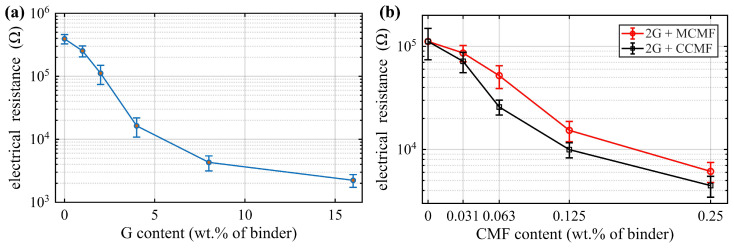
Percolation behavior at 28 days: (**a**) electrical resistance as a function of G content; and (**b**) electrical resistance as a function of MCMF and CCMF content (with 2 wt.% G).

Figure 6a presents the 28-day electrical resistance of G-filled specimens as a function of G content, with error bars representing the full range of values obtained from three replicate specimens. The results show that the electrical resistance decreases markedly with increasing G content, with the most pronounced reduction occurring between 2 wt.% and 4 wt.%. In this range, the resistance decreases from approximately $9.0\times 10^{4}\;\Omega$ to $1.5\times 10^{4}\;\Omega$, corresponding to a reduction of about 83%. Beyond this transition region, further increases in G content result in a more gradual decrease in resistance, indicating that a conductive network has largely been established. A content of 2 wt.% was selected to balance electrical performance and printability in this study. Increased G content raises water demand and reduces flowability, which can adversely affect extrusion stability and print quality.

The percolation behavior of mixtures containing 2 wt.% G combined with MCMF or CCMF is shown in Figure 6b. The addition of both CMF types further reduces the electrical resistance with respect to the 2G0CMF reference, confirming their effectiveness in enhancing conductive network formation. The CCMF-based mixtures exhibit a more pronounced reduction in resistance at low dosages, particularly between 0.03125 wt.% and 0.0625 wt.%, whereas the MCMF-based mixtures show a more gradual decrease across the investigated range. This difference is attributed to variations in fiber morphology and dispersion affecting conductive path formation. From these results, a combination of 2 wt.% G with 0.25 wt.% MCMF and 0.0625 wt.% CCMF was selected as the final mix design for printing the beam specimens among the investigated mixes, as higher CCMF contents increased viscosity and induce fiber entanglement resulting in nozzle blockage or unstable extrusion.

### 4.3. Mechanical Properties

**Figure 7:**
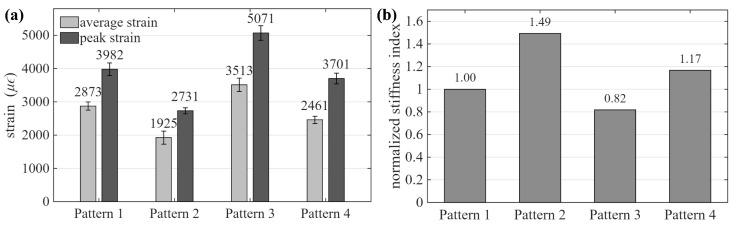
Comparison of (**a**) average strain and peak strain; and (**b**) normalized relative flexural stiffness index for the four beam configurations under cyclic three-point bending.

**Figure 8:**
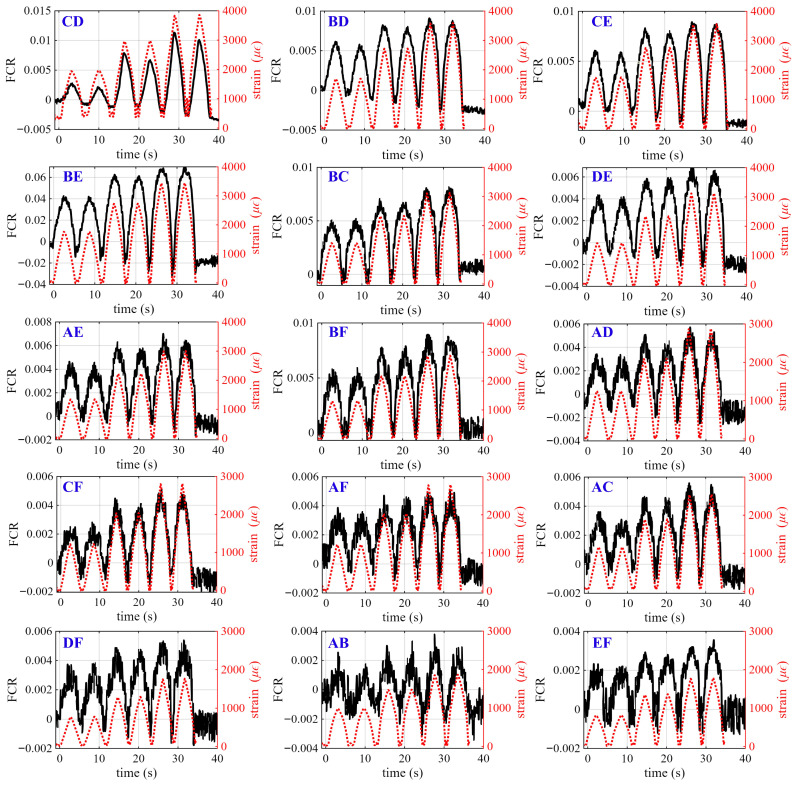
Time-series of FCR measured across electrode pairs for smart beam specimens—Pattern 1.

**Figure 9:**
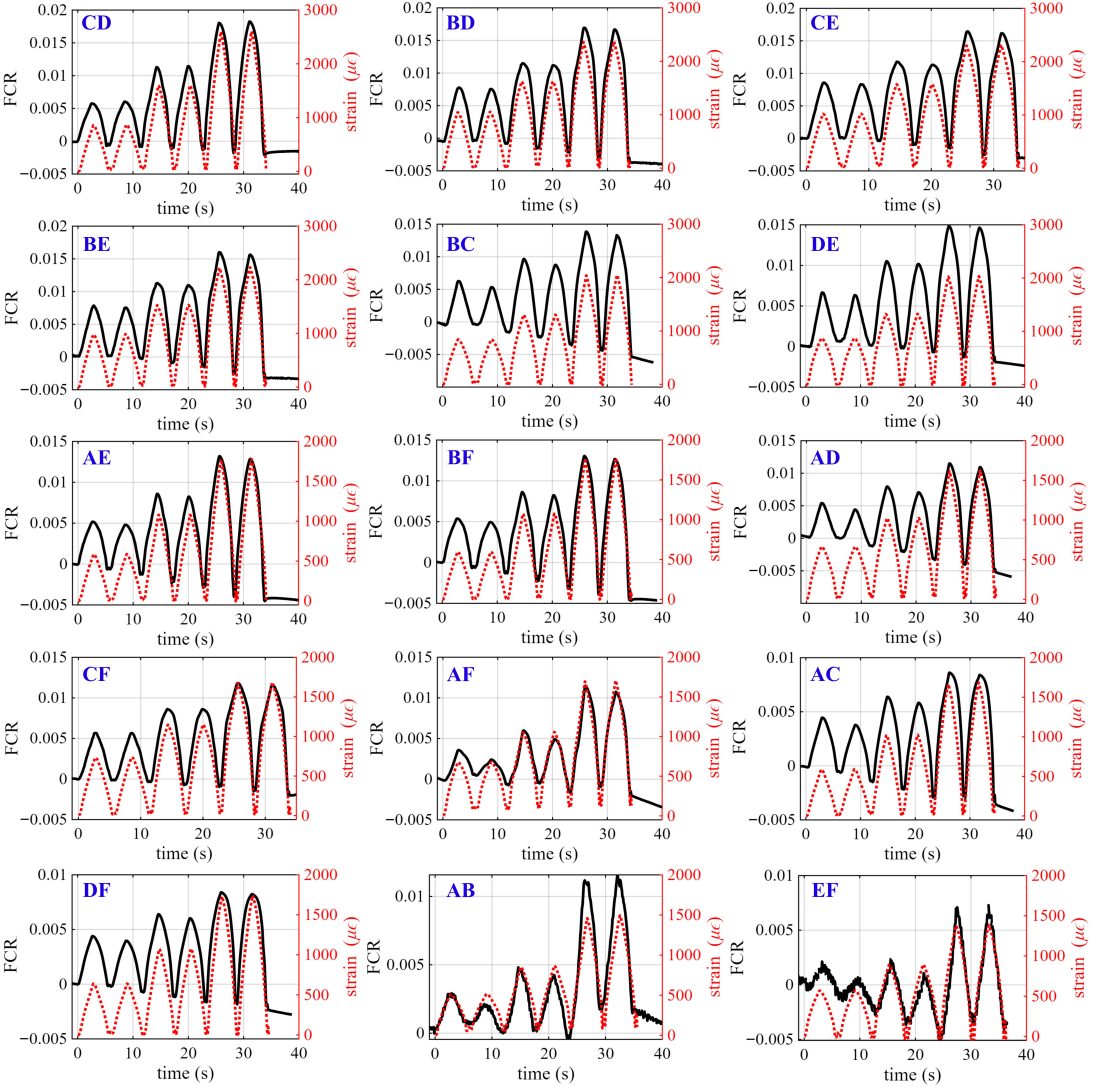
Time-series of FCR measured across electrode pairs for smart beam specimens—Pattern 2.

**Figure 10:**
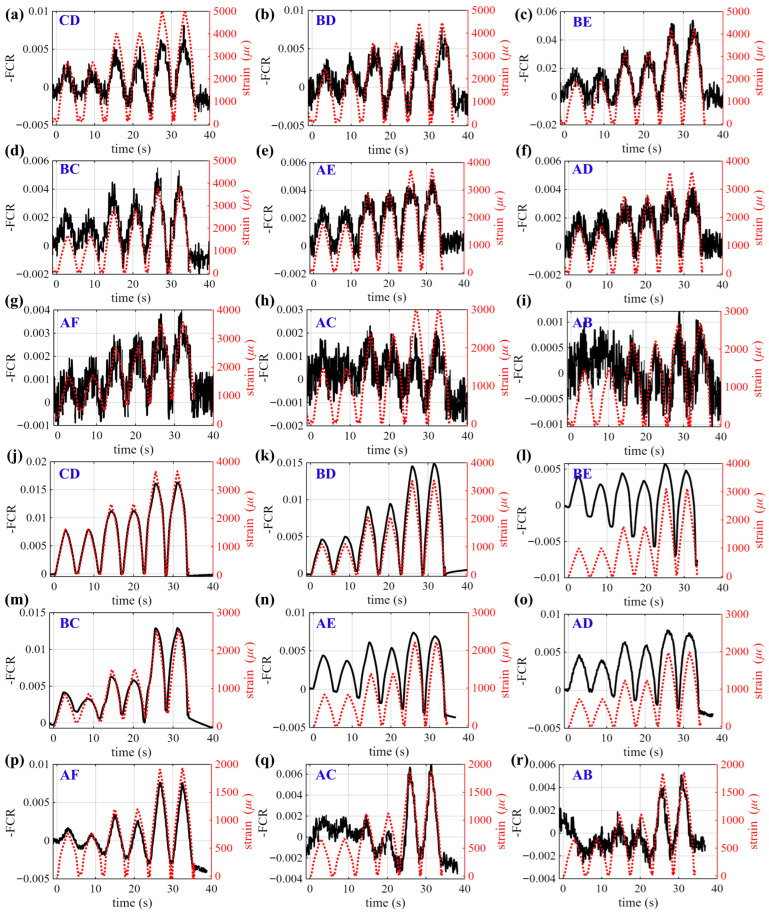
Time-series of -FCR measured across electrode pairs for smart beam specimens: (**a**–**i**) Pattern 3; and (**j**–**r**) Pattern 4. For symmetric electrode pairs (e.g., BD and CE), only one representative pair is shown.

The mechanical response of the four beam configurations was evaluated based on the strain data obtained during cyclic three-point bending, and results are presented in Figure 7. All specimens exhibited largely linear and repeatable load–displacement behavior across loading cycles, with minimal residual deformation upon unloading, indicating that the tests remained within the elastic regime, as shown in Figure 8, Figure 9 and Figure 10.

The average strain magnitudes of Patterns 1 to 4, obtained by averaging the bending strain across all electrode pairs, were 2873 με, 1925 με, 3513 με, and 2461 με, respectively. Patterns 3 and 4 exhibited 22.2% and 27.8% higher average strain compared to Patterns 1 and 2, respectively. This difference is attributed to the downward shift of the neutral axis caused by the bottom reinforcement and section geometry, which increases the distance between the neutral axis and the top conductive layer.

In addition to the average response, the peak strain values observed at the maximum loading level reached approximately 3982 με and 5071 με for Patterns 1 and 3, respectively, and 2731 με and 3701 με for Patterns 2 and 4, as shown in Figure 7a. These results further confirm that top-layer configurations (Patterns 3 and 4) experience higher strain amplitudes than bottom-layer configurations, while the inclusion of a solid-base region (Patterns 2 and 4) reduces overall deformation compared to their diagonal infill counterparts.

To further compare mechanical behaviors, a relative flexural stiffness index was defined based on the inverse relationship between bending strain and flexural rigidity under identical loading conditions. The index was computed using the average strain response obtained from the last three loading cycle (maximum load level) for each configuration. Specifically, the stiffness index for each configuration *p* is expressed as

(39) $$K^{\left(p\right)}\propto \frac{1}{{\bar{\varepsilon }}^{\left(p\right)}}$$

where ${\bar{\varepsilon }}^{\left(p\right)}$ denotes the average bending strain across all electrode pairs for pattern *p*. For comparison, the stiffness is normalized with respect to Pattern 1, yielding

(40) $${\tilde{K}}^{\left(p\right)}=\frac{{\bar{\varepsilon }}^{\left(1\right)}}{{\bar{\varepsilon }}^{\left(p\right)}}$$

The normalized stiffness values for Patterns 1 to 4 are 1.00, 1.49, 0.82, and 1.17, respectively, as shown in Figure 7b. This corresponds to increases of approximately 49.2% and 42.8% for Patterns 2 and 4 relative to their diagonal counterparts (Patterns 1 and 3), respectively, demonstrating that the incorporation of a solid-base region enhances load distribution and reduces deformation.

### 4.4. Strain Sensing

Figure 8 presents the time-series FCR responses measured between different electrode pairs for the beam specimen configured with Pattern 1, compared against the average bending strain computed from Equation (28) to Equation (33), during the dynamic test. Subplots show the electrode pair labeled at the top-left corner of each subplot. Black solid lines indicate the FCR response, while red dashed lines represent the corresponding strain input. The presented datasets are taken from the best-performing specimen, determined using the dynamic gauge factor ($\lambda_{\mathrm{dyn}}$). The presented data were post-processed using a Savitzky–Golay filter (first-order polynomial, window size of 100) to correct baseline drift associated with intrinsic polarization effects, which may otherwise affect the linearity of the FCR response. The window length was determined based on the sampling rate (10 S/s) and loading frequency (0.1 Hz), corresponding to approximately a full loading cycle. This configuration enabled effective noise reduction while preserving the amplitude and phase characteristics of the cyclic strain signal. Additional tests using window sizes of 80 and 120 resulted in variations in peak amplitude and gauge factor of less than 5%, indicating that the selected filtering parameters did not significantly influence the measured strain response.

The results show a close match between the electrical signal and strain time histories, with all electrode pairs exhibiting periodic FCR variations synchronized with the loading cycles. The amplitude of the FCR response varies almost systematically among electrode pairs, indicating the non-uniform bending strain distribution along the beam. In particular, electrode pairs located near the mid-span (e.g., CD, BD, and CE) exhibit the largest response amplitudes, whereas those located closer to the supports (e.g., AB and EF) show significantly smaller variations. This trend is consistent with the theoretical bending strain distribution (Equation (35)), in which the curvature, and thus strain, is maximized at the mid-span and decreases toward the supports. Intermediate electrode pairs (e.g., BC, DE, AE, and BF) exhibit responses of moderate amplitude, following the expected spatial variation in curvature.

Minor deviations from the ideal theoretical ranking (Equation (36)) are observed for certain electrode pairs, which can be attributed to localized variations in the conductive network at the mesoscale, slight variations in electrode contact conditions, and the inherent variability associated with additively manufactured cementitious materials. In addition, a certain degree of baseline drift and asymmetry between loading and unloading cycles is present in several signals, likely attributed to residual polarization effects (i.e., the time-dependent charge accumulation and relaxation at the electrode–matrix interface and within the cementitious matrix) and potential microstructural rearrangement under repeated loading. Additionally, extrusion-based 3D printing of cementitious materials may introduce interlayer defects and weak interfaces, which can influence both mechanical behavior and electrical response. In particular, interlayer bonding imperfections and shrinkage-induced microcracking may locally disrupt conductive pathways or alter strain transfer within the printed structure. In this study, all specimens were tested within the elastic regime, and no visible cracking or interlayer delamination was observed during loading. The consistent and repeatable piezoresistive responses across electrode pairs indicate that the conductive network remained stable under the applied conditions. However, the effects of printing-induced defects, including interlayer bonding imperfections and shrinkage-related cracking, as well as long-term performance and damage evolution, remain to be investigated in future work.

Figure 9 shows the corresponding FCR responses for the beam specimen configured with Pattern 2. Similary to Pattern 1, all electrode pairs exhibit cyclic variations that closely follow the applied strain, with response amplitudes varying along the beam length in accordance with the theoretical bending strain distribution (Equation (36)). This indicates that the relative distribution of bending strain along the beam is preserved regardless of internal pattern configuration. Compared to Pattern 1, the responses in Pattern 2 exhibit more uniform waveform shapes and reduced signal noise among cycles. This behavior is attributed to the presence of the solid base in Pattern 2, which enhances load transfer and promotes a more homogeneous strain field within the specimen. As a result, the electrical response becomes more stable and consistent across electrode pairs.

Figure 10 presents the time-series -FCR responses measured across representative electrode pairs for Patterns 3 and 4, where symmetric pairs are omitted for clarity. For these configurations, the conductive layer is located near the top of the beam and is therefore subjected to compressive strain under three-point bending. In contrast to the tensile configurations, the -FCR increases with increasing load, as compression promotes densification of the conductive network and enhances inter-particle contact. Despite the sign reversal, the spatial variation among electrode pairs remains consistent with the bending strain distribution, with signal amplitudes decreasing from the mid-span toward the supports.

### 4.5. Sensing Performance Metrics

**Figure 11:**
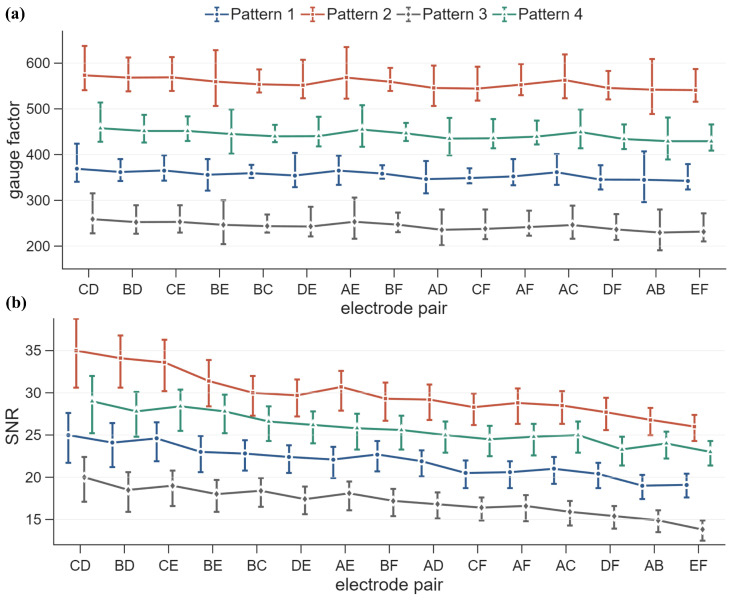
Comparison of three-specimen averaged sensing metrics: (**a**) gauge factor and (**b**) SNR, computed across electrode pairs for specimens under different configuration patterns.

Figure 11 presents the gauge factor and SNR across different electrode pair configurations for the four sensing patterns. The gauge factor was determined from cyclic loading data by analyzing the average power spectral density and calculated as the ratio between resistance variation and the corresponding strain response, while the SNR was computed using Equation (38). It is found that both metrics exhibit consistent spatial trends across electrode pairs, while clear differences arise from the electrode configurations and the placement of the conductive layers.

As shown in Figure 11a, the gauge factor remains relatively uniform across electrode pairs within each pattern, indicating the formation of a stable percolated conductive network. The corresponding standard deviations are 8.4, 10.9, 8.5, and 9.1 for Patterns 1 to 4, respectively. The average gauge factors are 355, 556, 244, and 443 for Patterns 1 to 4, respectively, which are significantly higher than typical values reported for graphite-based cementitious composites (generally below 200) [26,58]. This suggests enhanced electromechanical sensitivity, likely resulting from improved conductive network connectivity and the tailored structural configurations.

Among the four patterns, Pattern 2 exhibits the highest gauge factor, followed by Patterns 4, 1, and 3. In relative terms, Pattern 2 shows higher sensitivity than Patterns 1, 4, and 3 by 56.6%, 25.6%, and 128.1%, respectively. This enhanced response can be attributed to two primary factors. First, the conductive layer is located in the tensile region, where strain is maximized under bending, leading to greater perturbation of the conductive network. Second, the solid-base configuration promotes more continuous conductive pathways aligned with the current flow, enabling efficient translation of strain-induced changes in interparticle contacts into electrical signals. In contrast, Pattern 3 yields the lowest gauge factor due to less effective engagement of the conductive network.

A similar trend is observed when comparing patterns with an identical conductive-layer placement. Between Patterns 1 and 2 (bottom layer), Pattern 2 exhibits a 56.6% higher gauge factor, while between Patterns 3 and 4 (top layer), Pattern 4 shows a 97.8% increase over Pattern 3. This indicates that the presence of a solid base enhances sensing performance regardless of whether the conductive layer is in tension or compression. This effect is attributed to improved load transfer and a more homogeneous strain field, which stabilize the conductive network and reduce variability in the electrical response.

Comparing Patterns 1 and 3, which share the same diagonal infill but differ in conductive-layer placement, Pattern 1 exhibits a gauge factor approximately 45.5% higher than Pattern 3. This difference reflects the distinct electromechanical response under tension and compression. Under tensile loading, microcrack initiation and interparticle separation lead to more pronounced disruption of conductive pathways, resulting in larger resistance changes. In contrast, compressive loading tends to densify the conductive network and enhance particle contact, producing a more gradual response. Similar tension–compression asymmetry has been widely reported in self-sensing cementitious materials [26,59,60]. In addition to intrinsic material behavior, the presence of reinforcement may also influence the measured response by altering stress distribution and strain localization within the beam. Although not explicitly modeled in this study, this effect may contribute to the observed differences among configurations and is left for future investigation.

Figure 11b shows that the SNR consistently decreases from electrode pair CD to EF, which is associated with increasing current path length and accumulated electrical noise. Longer conduction paths result in higher resistance, reducing signal amplitude, while simultaneously increasing the influence of accumulated electrical noise and material heterogeneity. The reduction from CD to EF is about 25.7%, 20.7%, 23.6%, and 31.0% for Patterns 2, 4, 1, and 3, respectively. The SNR ranking follows the same order as the gauge factor (Figure 11a), indicating that sensitivity and signal stability are likely governed by the same mechanism. In particular, the average SNR of Pattern 2, which incorporates a solid base, is about 36.4% higher than that of Pattern 1, while Pattern 4, also featuring a solid base, exhibits an approximately 50.9% increase compared to Pattern 3. The results provides an insight that configurations with continuous conductive networks and stable structural support not only enhance resistance change but also reduce noise by stabilizing current distribution in additively manufactured components. The sensing performance, in terms of gauge factor, standard deviation, and SNR across different pattern configurations are summarized in Table 3.

## 5. Conclusions

This study investigated the performance of smart beam elements capable of self-sensing, demonstrating the feasibility of localizing sensing nodes within 3D-printed cementitious components for condition assessment and quality control. A Sikacrete-based composite incorporating G, MCMF, and CCMF was developed to achieve electrical percolation and a stable piezoresistive response, and implemented through a controlled multi-material printing strategy. The relative sensing performance of different structural configurations was investigated.

Electrical percolation analysis identified an optimal composition of 2 wt.% G, 0.25 wt.% MCMF, and 0.0625 wt.% CCMF, balancing conductivity and printability. This mixture was embedded within beam specimens in either tensile or compressive regions, combined with diagonal infill and solid-base configurations. Two internal structural configurations were considered, consisting of a fully patterned diagonal infill and a partially solid configuration with a 100% infill base. An electromechanical model was formulated to relate the fractional change in resistance to bending strain, accounting for variations in neutral-axis location, sensing-layer position, and electrode spacing, and enabling interpretation of the spatially distributed electrical measurements obtained during cyclic three-point bending.

Results show that all configurations exhibited consistent and repeatable piezoresistive responses, with electrical signals closely following the applied strain. The gauge factor remained relatively uniform within each configuration, while clear differences were observed among patterns. Specimens with sensing layers in the tensile region (Patterns 1 and 2) exhibited higher sensitivity than those in compression (Patterns 3 and 4). Pattern 2 exhibited the highest gauge factor ($\bar{\lambda }=556$), followed by Patterns 4, 1, and 3. The spatial variation of the response was consistent with the theoretical strain distribution along the beam, with normalized strain magnitudes ranging from 1.000 at the mid-span segment (CD), used as the reference, to 0.458 near the supports (e.g., AB and EF). Signal quality followed a similar pattern-level trend. Pattern 2 showed a 36.4% higher SNR than Pattern 1, while Pattern 4 exceeded Pattern 3 by 50.9%. In addition, SNR decreased with increasing electrode spacing, with reductions of 25.7%, 20.7%, 23.6%, and 31.0% for Patterns 2, 4, 1, and 3, respectively. Mechanical analysis indicated consistent elastic behavior across all configurations. The average strain values for Patterns 1 to 4 were 2873, 1925, 3513, and 2461 με, respectively.

Overall, this study demonstrated the feasibility of embedding self-sensing cementitious layers within structurally relevant 3D-printed elements using hybrid conductive composites. The proposed approach provided a practical pathway for integrating strain-sensing functionality directly into additively manufactured structures, enabling simultaneous in-print and in-operation structural health monitoring. The effect of damage evolution, long-term performance, environmental effects, and reinforcement effects on sensing behavior, as well as scalability to larger structural components, will be investigated in future work.

## Figures and Tables

**Table 1:** Material properties for the Fisher Chemical, APS 7–11 μm, 99% G powder and SGL milled and chopped CMF, adapted from [41].

Properties	G	MCMF	CCMF
density (g/cm^3^)	1.06	1.80	1.80
mean fiber length (μm)	-	150	6000
filament diameter (μm)	37	7	7
tensile strength (GPa)	-	4.0	4.0
tensile modulus (GPa)	-	240	240
elongation at break (%)	-	1.7	1.7
filament resistivity (μΩm)	40	15	15
bulk density (g/L)	800	250	-
sizing type	unsized	unsized	glycerin
sizing mass content (%)	-	-	4.0

**Table 2:** Mixture proportions for each type of 3DP conductive concrete specimens, with G/S (%) denoting the mass fraction of graphite relative to the Sikacrete binder.

Specimen	G (g)	G/S (%)	MCMF (g)	MCMF/S (%)	CCMF (g)	CCMF/S (%)	w/b (%)
0G0CMF	0	0	-	-	-	-	0.21
1G0CMF	3	1	-	-	-	-	0.23
2G0CMF	6	2	-	-	-	-	0.26
4G0CMF	12	4	-	-	-	-	0.29
8G0CMF	24	8	-	-	-	-	0.34
16G0CMF	48	16	-	-	-	-	0.44
2G31MCMF	6	2	0.09375	0.03125	-	-	0.26
2G62MCMF	6	2	0.1875	0.0625	-	-	0.26
2G125MCMF	6	2	0.375	0.125	-	-	0.26
2G250MCMF	6	2	0.75	0.250	-	-	0.26
2G31CCMF	6	2	-	-	0.09375	0.03125	0.26
2G62CCMF	6	2	-	-	0.1875	0.0625	0.26
2G125CCMF	6	2	-	-	0.375	0.125	0.26
2G250CCMF	6	2	-	-	0.75	0.250	0.26

**Table 3:** Summary of sensing performance, variability, and structural characteristics across different pattern configurations. Arrows indicate relative increasing (↑) or decreasing (↓) trends among the evaluated configurations.

Pattern	Configuration	$\bar{\lambda }$	Std	SNR Trend	Relative Performance
P1	bottom layer	355	8.4	↓ (23.6%)	baseline
P2	bottom + solid base	556	10.9	↓ (25.7%)	+56.4% $\bar{\lambda }$ vs. P1; +36.4% SNR
P3	top layer	244	8.5	↓ (31.0%)	lowest $\bar{\lambda }$
P4	top + solid base	443	9.1	↓ (20.7%)	+81.6% $\bar{\lambda }$ vs. P3; +50.9% SNR

## Data Availability

The data are available in a publicly accessible repository at the following link: https://www.dropbox.com/scl/fo/9lqctuls6xagyy2lm1dgb/APHe2cS5ESMJHpV6KTocFhM?rlkey=qz0ee0s7cggdkybuosp5idxbo&st=e27ty8ls&dl=0 (accessed on 2 May 2026).

## Abbreviations

The following abbreviations are used in this manuscript:

3DP	Three-dimensional printing
SHM	Structural health monitoring
G	Graphite
CMF	Carbon microfibers
MCMF	Milled carbon microfibers
CCMF	Chopped carbon microfibers
SEM	Scanning electron microscopy
w/b	Water-to-binder ratio
FCR	Fractional change in resistance
GF	Gauge factor
SNR	Signal-to-noise ratio
PSD	Power spectral density
LCR	Inductance-capacitance-resistance (meter)
R-X	Resistance-reactance (measurement mode)
AC	Alternating current
LVDT	Linear variable differential transformer
